# Case report: A variant of wall-eyed bilateral internuclear ophthalmoplegia from unilateral pons infarction

**DOI:** 10.3389/fnins.2022.974645

**Published:** 2022-09-09

**Authors:** Tingting Wang, Duanhua Cao, Jingzhe Han

**Affiliations:** Department of Neurology, Harrison International Peace Hospital, Hengshui, China

**Keywords:** wall-eyed internuclear ophthalmoplegia acute infarction, magnetic resonance imaging, pons, infarction, case

## Abstract

Wall-eyed bilateral internuclear ophthalmoplegia (WEBINO) is an uncommon ocular motility disorder that encompasses the following clinical signs: bilateral adduction deficits, bilateral abducting nystagmus, convergence lost, and a large angle exotropia in primary gaze. Here we report a case of a 55-year-old man presenting with atypical WEBINO syndrome with unilateral exotropia. The coverage test was used to record the patient's alternating exotropia. The patient experienced diplopia and ophthalmoplegia and was admitted to our hospital 3 days after the onset of the double vision. Neurologic examination showed left eye exotropia and bilateral internuclear ophthalmoplegia with impaired convergence. Vertical saccades of the left eye were also limited. Consequently, an MRI scan suggested an acute infarction in the left of the pontine tegmentum. The patient was finally diagnosed with pons infarction and was treated with anticoagulation and anti-platelet aggregation therapy.

## Case presentation

**Figure 1:**
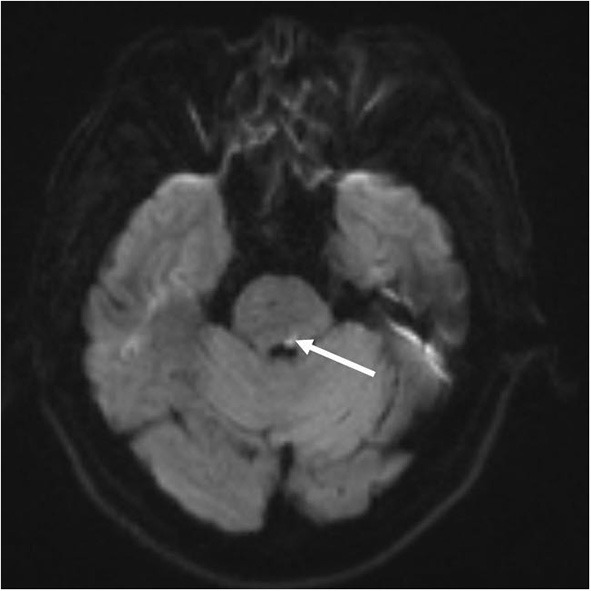
Brain MRI: Diffusion-weighted images reveal a focal ischemic stroke in the dorsal pons (arrows).

A 55-year-old man experienced diplopia and ophthalmoplegia and was admitted to our hospital 3 days after the onset of the double vision. He had a history of hypertension for more than 20 years. His highest blood pressure was 180/104 mmHg. Neurologic examination and cover test showed exotropia of the left eye, alternating exotropia, and bilateral internuclear ophthalmoplegia with impaired convergence. Vertical saccades and smooth pursuit of the left eye were also limited (see Supplementary Video). Pupillary, levator function, and bilateral abducent nucleus were normal. No other positive signs of the nervous system were found in physical examination. Besides, no abnormalities were found after liver and kidney function checks, blood tests, and the coagulation markers detection. Moreover, no abnormality was found in the levels of hepatitis B surface antigen (HbsAg), hepatitis B core antibody (HbcAb), hepatitis Be antigen (HBeAg), anti-hepatitis B e antibody (anti-HBe), anti-hepatitis B core antibody (anti-HBc), anti-hepatitis C virus (anti-HCV), anti-human immunodeficiency virus (anti-HIV), anti-treponema pallidum (anti-TP) antibody, anti-streptolysin O (ASO), rheumatoid factor (RF), C-reactive protein (CRP), and the autoantibodies. The lumbar puncture showed that the pressure was about 120 mmH2O, and the cerebrospinal fluid (CSF) analysis was normal. Consequently, the patient underwent an MRI scan, which suggests an acute infarction in the left of the pontine tegmentum involving the left medial longitudinal fasciculus (MLF) (Figure 1). The patient was finally diagnosed with pons infarction.

The patient received anticoagulation and anti-platelet aggregation therapy. There was no stroke recurrence, and diplopia returned to a healthy level after the patient's discharge from the hospital 6 months.

## Discussion

Wall-eyed bilateral internuclear ophthalmoplegia is a syndrome that involves bilateral adduction deficits, bilateral abducting nystagmus, and a large angle exotropia in primary gaze (Sharpe et al., 1974). WEBINO is mainly caused by cerebrovascular disorders (CVD), multiple sclerosis, neurodegenerative disorders, and infections (Sakamoto et al., 2012). WEBINO syndrome caused by CVD has been reported by several studies (Chen and Lin, 2007). Studies have shown that the disease is often associated with brainstem ischemia and hydrocephalus, immunotherapy of tumors, and diseases such as multiple sclerosis. This study summarized 39 case reports, including 42 WEBINO patients (Inocencio and Ballecer, 1985; Lana et al., 1990; Fay and Strominger, 1999; Korkmaz et al., 2002; Ozer et al., 2005; Chen and Lin, 2007; Kim et al., 2008; Matsumoto et al., 2008, 2019; Ushio et al., 2008; Jacob et al., 2010; Sierra-Hidalgo et al., 2010; Shinoda et al., 2011; Beh and Frohman, 2012; Jadhav and Prasad, 2012; Sakamoto et al., 2012; Bar et al., 2014; Chakravarthi et al., 2014; de Mora et al., 2014; Mathis et al., 2014; Muralidhar et al., 2014; Nakajima et al., 2014; Toufeeq and Dave, 2014; Agarwal et al., 2015; Ljevak et al., 2015; Man et al., 2015; de Souza et al., 2017; Sajjadi et al., 2017; Zou and Chen, 2017; Keereman et al., 2018; Papageorgiou et al., 2018; Im et al., 2020; Vázquez-Justes et al., 2020; Yazdi et al., 2020; Ansari et al., 2021; Jo et al., 2021; Petrik et al., 2021; Sinha et al., 2021; Wako et al., 2022). The age range of the patients was 12–85 years; 26 were male (61.9%), 15 were female (35.7%), and the gender of one was unknown. In the analysis of the clinical data of neuro-ophthalmological signs in WEBINO patients, 37 (88.1%) cases of exotropia in the first eye-position, 38 (90.5%) cases of bilateral intraocular disorders, 22 (52.4%) cases of convergence barrier, six (14.3%) cases of blepharoptosis, 18 (42.9%) cases of vertical gaze disorder, six (14.3%) cases of vertical nystagmus, eight (19.0%) cases of pupillary anomalies, and two (4.8%) cases of reversed dip. Among the studies, the lesion site summary showed that 28 (66.7%) cases occurred in the midbrain, 14 (33.3%) in the pons, nine (21.4%) in both the midbrain and the pons, and one (2.4%) in the brainstem. Among the causes of morbidity in WEBINO patients, 26 (61.9%) had cardiovascular and cerebrovascular diseases, ten (23.8%) neurological diseases, three (7.1%) infectious diseases, two (4.8%) tumor-related diseases, one (2.4%) alcohol overdose, and one (2.4%) drug-immune reaction. More details are shown in Table 1.

**Table 1:** Analysis of clinical data of WEBINO patients.

**Case (references)**	**Patient**	**Age**	**Gender**	**Neuro-ocular signs**								**Etiology**	**Occlusion site**
				**Aa**	**Bb**	**Cc**	**Dd**	**Ee**	**Ff**	**Gg**	**Hh**		
1. Inocencio and Ballecer (1985)	1	24	M	Y	Y	Y	N	N	N	N	N	CNS MTI	Mid, Pons
2. Lana et al. (1990)	1	35	F	Y	Y	Y	N	N	N	N	N	CNS vasculitis	–
3. Fay and Strominger (1999)	1	33	M	Y	Y	Y	N	N	N	N	N	CNS Cry	Mid
4. Korkmaz et al. (2002)	1	14	F	Y	Y	N	N	N	N	N	N	CIDP	–
5. Ozer et al. (2005)	1	15	M	Y	–	–	–	–	–	Y	N	DAD	Mid, Pons
6. Chen and Lin (2007)	4	66	M	Y	Y	N	N	Y	N	N	N	CI	Mid
		84	M	Y	Y	N	N	Y	N	N	N	CI	Mid, Pons
		51	F	Y	Y	N	N	N	N	N	N	Hyd, brainstem neoplasms	Mid, Pons
		65	F	Y	Y	Y	N	N	N	N	N	CI	Pons
7. Kim et al. (2008)	1	78	M	Y	Y	Y	N	Y	N	N	N	CI	Mid
8. Matsumoto et al. (2008)	1	72	M	Y	Y	Y	N	Y	N	N	N	PSP	Mid
9. Ushio et al. (2008)	1	72	M	Y	Y	Y	N	Y	N	N	N	PSP	Mid
10. Jacob et al. (2010)	1	67	M	Y	–	–	–	–	N	N	N	Hyd, SAH	–
11. Sierra-Hidalgo et al. (2010)	1	55	F	Y	Y	N	Y	Y	N	Y	N	CI	Mid
12. Shinoda et al. (2011)	1	19	F	Y	Y	N	N	N	N	Y	N	NMOSD	Mid
13. Beh and Frohman (2012)	1	69	M	Y	Y	Y	N	Y	N	N	N	CI	Mid, Pons
14. Jadhav and Prasad (2012)	1	41	M	Y	Y	N	N	N	Y	N	N	Hyd, CM	–
15. Sakamoto et al. (2012)	1	64	M	Y	Y	Y	N	N	N	N	N	CI	Pons
16. Bar et al. (2014)	1	12	F	Y	Y	N	N	Y	Y	N	N	Dem	Mid
17. Chakravarthi et al. (2014)	1	64	F	Y	Y	Y	N	Y	N	N	N	CI	Mid
18. Mathis et al. (2014)	1	68	M	Y	Y	Y	N	N	N	N	N	CI	Pons
19. Muralidhar et al. (2014)	1	35	M	Y	Y	N	N	N	N	N	N	Alcohol	-
20. Nakajima et al. (2014)	1	68	M	Y	Y	Y	Y	Y	N	N	N	CI	Mid, Pons
21. de Mora et al. (2014)	1	57	M	Y	Y	Y	N	N	Y	N	N	CI, Neurosyphilis	Mid
22. Toufeeq and Dave (2014)	1	13	M	Y	Y	Y	N	Y	Y	Y	N	Pineal tumor	Mid
23. Agarwal et al. (2015)	1	19	F	Y	Y	Y	N	Y	Y	N	Y	CI	Mid, Pons
24. Ljevak et al. (2015)	1	53	M	Y	Y	N	Y	Y	N	Y	Y	CI	Mid
25. Man et al. (2015)	1	84	M	Y	Y	Y	N	Y	N	N	N	CI	Mid
26. de Souza et al. (2017)	1	60	F	Y	Y	Y	N	Y	N	Y	N	PSP	Mid
27. Sajjadi et al. (2017)	1	38	M	Y	Y	Y	N	N	N	N	N	–	–
28. Zou and Chen, 2017)	1	70	F	Y	Y	Y	Y	Y	N	N	N	NMOSD	Mid
29. Papageorgiou et al. (2018)	1	65	M	Y	Y	N	N	N	N	N	N	CI	Mid
30. Keereman et al. (2018)	1	25	F	Y	Y	N	Y	N	N	Y	N	Hyd	–
31. Matsumoto et al. (2019)	1	81	M	N	Y	Y	N	Y	N	N	N	PSP	Mid
32. Yazdi et al. (2020)	1	57	M	Y	Y	Y	N	Y	N	N	N	PSP	Mid, Pons
33. Vázquez-Justes et al. (2020)	1	68	M	N	Y	N	N	N	N	N	N	Ischemic lesion	Mid
34. Im et al. (2020)	1	62	F	N	Y	N	N	N	N	N	N	Stroke	Pons
35. Petrik et al. (2021)	1	55	M	Y	N	N	N	N	N	N	N	CH	Pons
36. Jo et al. (2021)	1	–	–	–	–	–	–	–	–	–	–	CI	Mid
37. Sinha et al. (2021)	1	48	F	N	Y	N	Y	N	Y	Y	N	CH	Brainstem
38. Ansari et al. (2021)	1	45	F	Y	Y	N	N	N	N	N	N	irEA	–
39. Wako et al. (2022)	1	85	M	Y	Y	Y	N	N	N	N	N	CES	Mid, Pons, Cer

Aa, Exotropia in the first eye-position; Bb, Bilateral Intraocular Disorders; Cc, Convergence barrier; Dd, Blepharoptosis; Ee, Vertical gaze disorder; Ff, Vertical nystagmus; Gg, Pupillary anomalies; Hh, Reversed dip. M, Male; F, Female; Y, yes; N, No; CI, Cerebral infarction; CH, Cerebral hemorrhage; Mid, Midbrain; Cer, cerebellum; Cry, Cryptococcosis infection; MTI, Mycobacterium tuberculosis infection; CNS, Central nervous system; CIDP, Chronic inflammatory demyelinating polyradiculoneuropathy; Hyd, Hydrocephalus; DAD, Diffuse axonal damage; PSP, Progressive superanuclear palsy; CES, cardioembolic stroke; SAH, Subarachnoid hemorrhage; NMOSD, Neuromyelitis optica spectrum disorders; CM, Cryptococcal meningitis; Dem, Demyelinating Diseases; irEA, Immune related adverse event.

Here we reported a single case of a patient with atypical WEBINO with unilateral exotropia (Supplementary Video) as the main clinical manifestation. The dissociated abducting nystagmus, impaired convergence, and supranuclear vertical gaze palsy implied a lesion of bilateral MLF. Yet, most of the infarcts in the left median dorsal pons cause unilateral, not bilateral, lesion of MLF (Gossman, 2006). The mechanism for the atypical clinical presentation in this patient may be due to the proximity of the unilateral lesion to the midline resulting in bilateral MLF lesions combined with converging fiber involvement resulting in WEBINO. Still, the causal pathophysiology remains unclear and disputed.

## Data availability statement

The original contributions presented in the study are included in the article/Supplementary material, further inquiries can be directed to the corresponding author/s.

## Ethics statement

The studies involving human participants were reviewed and approved by the Ethics Committee of Harrison International Peace Hospital. The patients/participants provided their written informed consent to participate in this study. Written informed consent was obtained from the individual(s) for the publication of any potentially identifiable images or data included in this article.

## Author contributions

JH and TW organized and proofread the writing of the manuscript. DC and TW wrote the manuscript draft. All authors contributed to the article and approved the submitted version.

## Conflict of interest

The authors declare that the research was conducted in the absence of any commercial or financial relationships that could be construed as a potential conflict of interest.

## Publisher's note

All claims expressed in this article are solely those of the authors and do not necessarily represent those of their affiliated organizations, or those of the publisher, the editors and the reviewers. Any product that may be evaluated in this article, or claim that may be made by its manufacturer, is not guaranteed or endorsed by the publisher.
